# Wearable sensor–derived gait signatures for Parkinson's disease: an exploratory diagnostic study integrating dimensionality reduction and machine learning

**DOI:** 10.3389/fmed.2026.1939598

**Published:** 2026-10-08

**Authors:** Guo Hong, Xueyi Wen, Jinggui Gao, Jiayan Lin, Shawutijiang Zumulaiti, Yunzhu Yang, Xiaohua Xiao

**Affiliations:** 1 Department of Geriatrics, Shenzhen Second People’s Hospital (The First Affiliated Hospital of Shenzhen University), Shenzhen, China; 2 Department of Neurology, Shenzhen People’s Hospital (The Second Clinical Medical College, Jinan University; The First Affiliated Hospital, Southern University of Science and Technology), Shenzhen, China; 3 Department of Neurology, Shenzhen Second People’s Hospital (The First Affiliated Hospital of Shenzhen University), Shenzhen, China

**Keywords:** gait analysis, machine learning, Parkinson’s disease, principal component analysis, wearable sensors

## Abstract

**Background:**

Parkinson's disease (PD) is characterized by progressive motor dysfunction, in which gait disturbances often emerge early yet remain underutilized in diagnostic frameworks. Advances in wearable sensor technology enable high-resolution quantification of gait dynamics, but the clinical translation of such high-dimensional data is limited by redundancy and model overfitting.

**Objective:**

To determine the exploratory diagnostic efficacy of synthesizing wearable sensor-derived gait parameters, cognitive scores, and blood biomarkers utilizing a dual-stage dimensionality reduction framework coupled with logistic regression machine learning for the precise identification of PD.

**Methods:**

In this cross-sectional study, demographic characteristics, blood biomarkers, cognitive assessments, and wearable sensor–derived gait parameters were collected from patients with PD and matched controls. Following initial group comparisons, least absolute shrinkage and selection operator (LASSO) regression was applied to identify candidate predictors from multimodal variables. Principal component analysis (PCA) was subsequently performed to reduce dimensionality and extract latent structures. Logistic regression models were constructed using both the original LASSO-selected variables and PCA-derived components. Model performance was assessed using 10-fold cross-validation, with discrimination quantified by the area under the receiver operating characteristic curve (AUC) and accuracy.

**Results:**

Baseline characteristics were comparable between groups except for age. LASSO identified 22 potential predictors spanning demographic, biochemical, cognitive, and gait domains. PCA further condensed these variables into six principal components, explaining 62.983% of the total variance. Using a rigorous nested cross-validation framework on the primary cohort, the PCA-based model achieved an internal mean AUC of 0.949 with an accuracy of 0.827. Furthermore, when evaluated on a completely independent external cohort (*N* = 36) to ensure generalizability, the finalized PCA-based model demonstrated robust diagnostic performance, yielding an AUC of 0.891 and an accuracy of 0.750. Both approaches showed robust discriminative capacity, with dimensionality reduction enhancing overall efficiency and stability.

**Conclusion:**

Integrating wearable gait signatures with LASSO-PCA dimensionality reduction and logistic regression yields reliable, interpretable PD classification, with PCA-condensed features demonstrating enhanced diagnostic efficiency. These findings warrant external validation in multicentre, longitudinal cohorts to advance non-invasive, sensor-based PD screening.

## 1 Introduction

Parkinson's disease (PD) is a complex neurodegenerative syndrome driven by heterogeneous pathophysiological mechanisms, where early clinical manifestations often present as subtle, multifaceted gait and postural dysfunctions (1, 2). Standard clinical diagnostic frameworks, while foundational to neurological practice, predominantly rely on qualitative, episodic evaluations that can easily overlook the micro-kinematic anomalies indicative of prodromal or early-stage disease (3). Consequently, translating precise kinematic profiles into objective digital biomarkers has emerged as a critical clinical imperative. Wearable inertial sensors enable the high-resolution, continuous capture of dynamic motor parameters, providing a robust, non-invasive methodology for tracking disease-specific motor phenotypes in naturalistic settings (4). Yet, integrating these devices into routine diagnostic workflows remains heavily encumbered by the profound multidimensionality of the resulting data, which often precipitates multi-collinearity, exacerbates algorithmic noise, and inflates the risk of overfitting during predictive modeling (5, 6).

Navigating this high-dimensional data environment requires mathematically rigorous dimensionality reduction prior to the application of predictive classification algorithms. Techniques such as the Least Absolute Shrinkage and Selection Operator (LASSO) apply strict L1 regularization to penalize redundant variables, ensuring that only the most sensitive multimodal risk factors are retained (7). Concurrently, Principal Component Analysis (PCA) orthogonally transforms these high-density matrices into concise, independent principal components, preserving essential clinical variance while radically diminishing computational burden (8). When coupled with robust machine learning classifiers, such as logistic regression, these integrated processing pipelines demonstrate immense potential to isolate pathological signatures from overly complex clinical datasets.

Despite these theoretical advances, a unified diagnostic framework successfully synthesizing serological variables, cognitive indices, and wearable-derived gait parameters through PCA and machine learning remains underexplored in current literature. Thus, this study designed an exploratory diagnostic model synthesizing comprehensive multimodal data. We hypothesized that sequentially integrating LASSO feature selection with PCA-derived dimensionality reduction would fundamentally optimize logistic machine learning frameworks. By comparatively evaluating the area under the receiver operating characteristic curve (AUC) of original vs. PCA-transformed metrics, this study aims to validate a highly efficacious, digital-forward diagnostic pipeline for PD, offering a scalable tool for early clinical intervention.

## 2 Materials and methods

### 2.1 Study population

A total of 177 patients with clinically diagnosed Parkinson's disease (PD) were consecutively enrolled from the inpatient and outpatient clinics of the Department of Neurology, Shenzhen People's Hospital (The Second Clinical Medical College of Jinan University; The First Affiliated Hospital of Southern University of Science and Technology), Shenzhen, Guangdong Province, China, between April 2023 and November 2024. In parallel, 63 age-matched healthy controls were recruited from the local community during the same period.

To explicitly prevent information leakage and rigorously validate the generalization capacity of our framework, an additional, entirely independent external validation cohort of 36 participants (comprising 25 patients with PD and 11 matched healthy controls) was subsequently recruited. Importantly, these 36 individuals were completely isolated from all exploratory phases of feature selection (LASSO) and dimensionality reduction (PCA), and were utilized exclusively for the final testing of the predictive machine learning models. All participants in this external cohort were subject to the exact same rigorous inclusion and exclusion criteria detailed below.

#### 2.1.1 Inclusion and exclusion criteria for PD patients

Inclusion Criteria: (1) Diagnosis of PD confirmed independently by two movement disorder specialists according to the Movement Disorder Society (MDS) Clinical Diagnostic Criteria (9); (2) Age ≥ 18 years; (3) Ability to walk independently without assistance; (4) Availability of complete and reliable clinical data; (5) Capacity to provide written informed consent voluntarily.

Exclusion Criteria: (1) History of other neurological or psychiatric disorders that may confound the diagnosis or management of PD; (2) History of deep brain stimulation (DBS) or other invasive intracranial surgical procedures; (3) Current use of medications known to interfere with PD symptomatology (e.g., dopamine receptor antagonists); (4) Severe systemic comorbidities involving the cardiovascular, hepatic, or renal systems; symptomatic orthostatic hypotension; or gait disorders attributable to causes other than PD; (5) History of substance abuse or alcohol use disorder; (6) Any other medical conditions deemed likely to influence the study outcomes.

#### 2.1.2 Inclusion and exclusion criteria for healthy controls

Inclusion criteria: (1) age ≥18 years; (2) availability of complete and reliable clinical data matched to those of the PD group; and (3) capacity to provide informed consent.

Exclusion criteria: (1) a history of neurological or psychiatric disorders; (2) use of medications that could interfere with motor function; (3) any abnormal findings on brain imaging or neurological examination; and (4) the presence of other medical conditions that could potentially affect the study outcomes.

### 2.2 Collection of clinical and demographic data

Clinical and demographic data were systematically collected for all enrolled participants, including: (1) Demographic characteristics: Age, Sex, Years of Education, Smoking status, and Alcohol drinking status; (2) Medical history: Hypertension, Diabetes, Hyperlipidemia, AF, and CHD.

### 2.3 Post-enrollment assessments and measurements

(1) Venous blood samples were collected from all enrolled participants at the outpatient clinic or during hospitalization. Routine laboratory parameters were measured, including WBC count, RBC count, PLT count, ALT, AST, BG, Creatinine, LDL, TG, HDL, and TC; (2) Cognitive function was assessed using the Mini-Mental State Examination (MMSE) and the Montreal Cognitive Assessment (MOCA); (3) Gait data were acquired from all participants using a wearable sensor-based gait analysis system; (4) This study was conducted in accordance with the ethical principles outlined in the *Ethical Review Measures for Biomedical Research Involving Human Subjects* (National Health Commission of the People's Republic of China, 2016), the *Declaration of Helsinki* (World Medical Association), and the *International Ethical Guidelines for Health-Related Research Involving Humans* (Council for International Organizations of Medical Sciences). The study protocol was approved by the Institutional Ethics Committee of Shenzhen People’s Hospital (approval no. LL-KY-2023175-02).

### 2.4 Wearable sensor-based gait analysis system: device description, pre-test preparation, and testing protocol

In this study, gait and motion quantification were performed using the MATRIX wearable assessment system (GYENNO SCIENCE, Shenzhen, China), a commercially available device (10). The system has received regulatory approval from the National Medical Products Administration (NMPA, China), the Food and Drug Administration (FDA, United States), and Conformité Européenne (CE) Medical certification. All participants were equipped with 10 inertial measurement unit (IMU) sensors operating at a sampling rate of 100 Hz (Figure 1A). Each IMU comprised a triaxial accelerometer (range: ±16 g; sensitivity: 16,384 LSB/g) and a triaxial gyroscope (range: ±2,000°/s; sensitivity: 131 LSB/°/s). Foot sensors were placed on the dorsal aspect of each foot over the metatarsal region. Bilateral thigh sensors were affixed approximately 2 cm superior to the patella, and bilateral shank sensors were positioned approximately 2 cm superior to the lateral malleolus. Bilateral hand sensors were secured on the dorsal aspect of each wrist. The chest sensor was anchored at the sternal level, and the lumbar sensor was positioned at the level of the fifth lumbar vertebra (L5). All sensors were fastened securely to their designated anatomical locations using adjustable straps (Figure 1B). Participants were instructed to walk back and forth along a straight, level corridor (8 m per pass) at their self-selected comfortable speed under natural conditions (Figure 1C). Patients with PD were assessed during the defined “ON” medication state. During walking, raw motion signals from the 10 wearable sensors were captured in real time and transmitted via Bluetooth to a host computer for subsequent analysis. Gait and postural transition parameters were automatically extracted from the raw motion signals using a pre-built algorithm implemented in MATLAB (MathWorks, Natick, MA, USA) (10).

**Figure 1:**
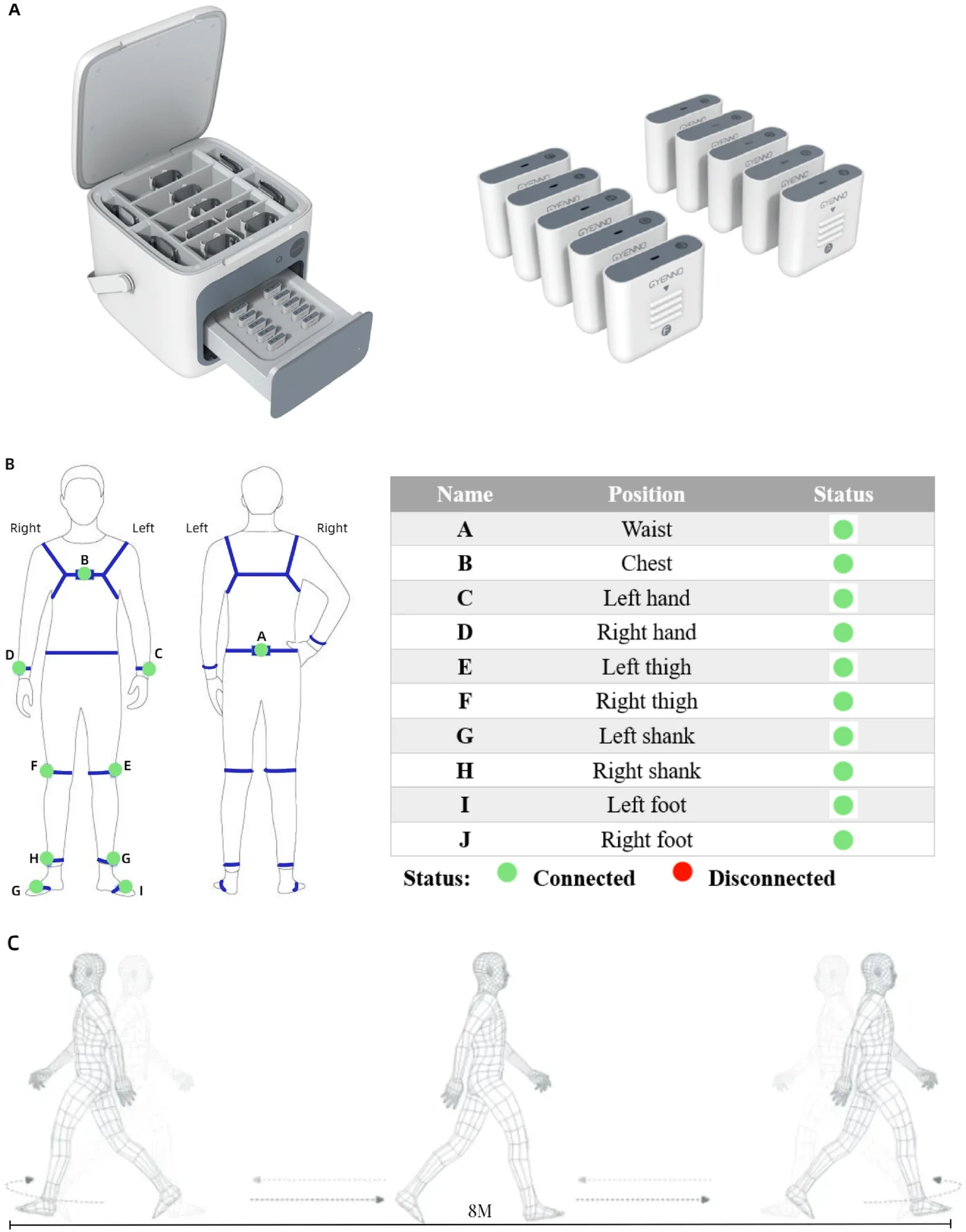
Schematic illustration of the wearable sensor-based gait analysis system and walking test protocol. **(A)** Wearable sensor storage box and ten sensor modules. **(B)** Sensor placement and connection status: A, waist; B, chest; C, left hand; D, right hand; E, left thigh; F, right thigh; G, left shank; H, right shank; I, left foot; and J, right foot. Green dots indicate connected sensors. **(C)** Schematic illustration of the 8-m straight walking test.

A total of 24 gait parameters were collected, comprising: step length, walk speed, stride length, stride time, cadence, double support, swing, stance, peak shank angular velocity, shank symmetry index, relative mean phase difference, phase coordination index, interlimb coordination, peak trunk coronal angular velocity, peak trunk sagittal angular velocity, peak trunk transverse angular velocity, peak trunk sway angle, peak arm angular velocity, arm symmetry index, average steering duration, average number of steps during steering, peak angular velocity during steering, average angular velocity during steering, and average time per step during steering.

### 2.5 Statistical analysis

Statistical analyses were performed using SPSS (version 27.0), R (version 3.6.1), and Python (version 3.9.12). Continuous variables are presented as the median and interquartile range (IQR) and were compared using the Mann–Whitney *U* test. Categorical variables are expressed as counts and percentages and were compared using the chi-square test.

The analytical pipeline was divided into two distinct phases to prevent information leakage: an exploratory descriptive phase and a predictive machine learning phase. (1) Exploratory Descriptive Phase: To map the global clinical and kinematic landscape of the primary cohort (*N* = 240) for descriptive visualization purposes only (e.g., generating global scatter plots and heatmaps), Least Absolute Shrinkage and Selection Operator (LASSO) regression was initially applied for feature selection on the entire dataset, followed by Principal Component Analysis (PCA) to extract latent clinical structures. The statistical weights derived from this global phase were strictly isolated and discarded prior to any predictive modeling. (2) Leakage-Free Predictive Machine Learning Phase: To rigorously evaluate diagnostic performance without information leakage, a nested machine learning workflow was employed. We introduced an additional, completely independent cohort of 36 participants (who were entirely excluded from the aforementioned exploratory phase). Logistic regression models were constructed using a stratified Nested Cross-Validation approach. During this process, all continuous variables underwent strict standard scaling (*z*-score normalization with zero mean and unit variance); subsequently, LASSO selection and PCA transformation were recalculate strictly within the training folds, ensuring that the internal validation data remained entirely blind to feature selection and dimensionality reduction. To systematically benchmark diagnostic performance, seven machine-learning algorithms were compared within the nested cross-validation framework using both the 22 LASSO-selected variables and six PCA-derived components. The independent external cohort (*N* = 36) was strictly excluded from model selection and reserved exclusively for final validation to prevent information leakage and selection bias. The diagnostic performance of the Clinical-Only Baseline, original-variable, and PCA-based models was comparatively evaluated using nested cross-validation in the primary cohort to assess the incremental value of wearable-derived gait features and PCA-based dimensionality reduction. Additionally, SHapley Additive exPlanations (SHAP) analysis was employed to quantify individual feature contributions and enhance the clinical interpretability of the predictive models. Finally, the finalized models were tested on the completely unseen external cohort of 36 individuals. Model generalizability and feature-selection robustness were further assessed in an independent external cohort (*N* = 36), providing an unbiased evaluation beyond internal resampling. All statistical tests were two-sided, with a significance threshold of *α* = 0.05.

## 3 Results

### 3.1 Comparison of baseline characteristics between the Parkinson's disease and healthy control groups

A total of 240 participants were enrolled in this study, including 63 individuals in the healthy control (HC) group and 177 patients in the Parkinson's disease (PD) group. Except for Age, which differed significantly between the two groups (*P* < 0.05), no statistically significant differences were observed in the remaining baseline characteristics, including Sex, Smoking, Alcohol drinking, Hypertension, Diabetes, Hyperlipidemia, AF, CHD, and Years of Education (all *P* > 0.05). Detailed results are presented in Table 1.

**Table 1:** Comparison of clinical baseline data between the HC and PD groups.

Variables	Category item	Total (*n* = 240)	HC (*n* = 63)	PD (*n* = 177)	*Z*	*P*
Sex, *n* (%)	Male	171 (71.25)	44 (69.84)	127 (71.75)	0.083	0.774
	Female	69 (28.75)	19 (30.16)	50 (28.25)		
Smoking, *n* (%)	NO	181 (75.42)	46 (73.02)	135 (76.27)	0.266	0.606
	YES	59 (24.58)	17 (26.98)	42 (23.73)		
Alcohol drinking, *n* (%)	NO	170 (70.83)	40 (63.49)	130 (73.45)	2.228	0.135
	YES	70 (29.17)	23 (36.51)	47 (26.56)		
Hypertension, *n* (%)	NO	147 (61.25)	41 (65.08)	106 (59.89)	0.528	0.468
	YES	93 (38.75)	22 (34.92)	71 (40.11)		
Diabetes, *n* (%)	NO	215 (89.58)	53 (84.13)	162 (91.53)	2.725	0.099
	YES	25 (10.42)	10 (15.87)	15 (8.48)		
Hyperlipidemia, *n* (%)	NO	164 (68.33)	42 (66.67)	122 (68.93)	0.110	0.741
	YES	76 (31.67)	21 (33.33)	55 (31.07)		
AF, *n* (%)	NO	221 (92.08)	57 (90.48)	164 (92.66)	0.303	0.582
	YES	19 (7.92)	6 (9.52)	13 (7.35)		
CHD, *n* (%)	NO	220 (91.67)	57 (90.48)	163 (92.09)	0.158	0.691
	YES	20 (8.33)	6 (9.52)	14 (7.91)		
Age (years)		62.00 [54.00, 67.00]	57.00 [53.00, 66.00]	63.00 [57.00, 67.00]	−2.207	0.027
Years of education (years)		12.00 [10.00, 15.00]	12.00 [12.00, 16.00]	12.00 [10.00, 15.00]	1.082	0.273
Disease duration (years)		N/A	N/A	3.00 [3.00, 5.00]		
MDS-UPDRS III		N/A	N/A	27.00 [22.00, 37.00]		
LEDD (mg/day)		N/A	N/A	435.00 [400.00, 510.00]		
H&Y1–2	NO	N/A	N/A	27 (15.25)		
	YES	N/A	N/A	150 (84.75)		

AF, atrial fibrillation; CHD, coronary heart disease; UPDRS-III, Unified Parkinson’s disease rating scale part III; LEDD, levodopa equivalent daily dose; H&Y, Hoehn and Yahr stage.

### 3.2 Screening of risk factors for Parkinson's disease

LASSO regression analysis was performed using the presence or absence of Parkinson's disease as the dependent variable and all remaining variables as independent variables (Figure 2). LASSO regression shrinks variable coefficients, thereby reducing overfitting and addressing severe multicollinearity. The results demonstrated that, at the optimal penalty parameter (*λ* = 0.005) corresponding to the minimum mean squared error, the number of variables was reduced from 38 to 22 (coefficients are presented in Table 2). The selected variables included WBC, PLT, ALT, AST, Creatinine, HDL, TC, MOCA, stride length, peak shank angular velocity, shank symmetry index, relative mean phase difference, interlimb coordination, peak trunk coronal angular velocity, peak trunk sagittal angular velocity, peak trunk sway angle, peak arm angular velocity, arm symmetry index, average number of steps during steering, peak angular velocity during steering, average angular velocity during steering, and average time per step during steering. Spearman correlation analysis showed reduced inter-variable redundancy after LASSO selection, with fewer highly correlated feature clusters among the 22 retained variables compared with the initial 38 candidates (Supplementary Figures S2, S3). This reduction in collinearity supported subsequent dimensionality reduction by PCA.

**Figure 2:**
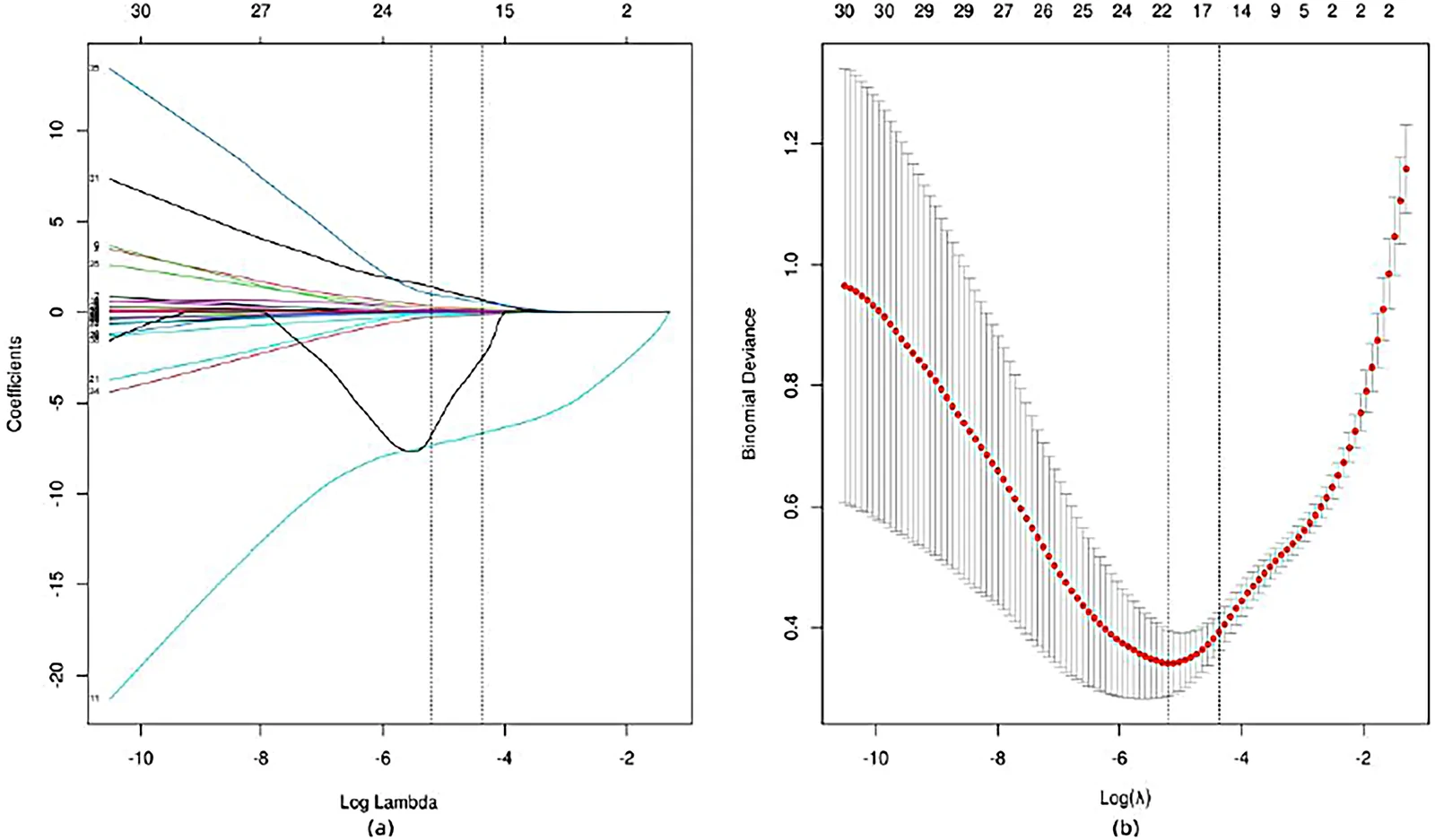
Feature selection using LASSO regression analysis. **(a)** Ten-fold cross-validation was used to determine the optimal penalty parameter, with the vertical line indicating the selected value of lambda that yielded 22 non-zero coefficients. **(b)** Coefficient profiles of the 38 features were plotted against the log(*λ*) sequence in the LASSO model. The vertical dashed lines indicate the lambda values corresponding to the minimum mean squared error (*λ* = 0.005) and the one-standard-error criterion (*λ* = 0.013), respectively.

**Table 2:** Lasso coefficient table.

Name	Minimum mean square error coefficient (*λ* = 0.005)	The one-standard-error criterion coefficient (*λ* = 0.013)
(Intercept)	20.047	15.338
Age	0.000	0.000
WBC	0.351	0.166
RBC	0.000	0.000
PLT	−0.003	0.000
ALT	0.052	0.027
AST	0.113	0.069
BG	0.000	0.000
Creatinine	0.012	0.000
LDL	0.000	0.000
TG	0.000	0.000
HDL	−7.327	−6.642
TC	0.200	0.091
MOCA	−0.075	0.000
MMSE	0.000	0.000
Step length	0.000	0.000
Walk speed	0.000	0.000
Stride length	−0.015	0.000
Stride time	0.000	0.000
Cadence	0.000	0.000
Double support	0.000	0.000
Swing	0.000	0.000
Stance	0.000	0.000
Peak shank angular velocity	0.015	0.002
Shank symmetry index	−0.291	−0.144
Relative mean phase difference	0.214	0.131
Phase coordination index	0.000	0.000
Interlimb coordination	−0.007	0.000
Peak trunk coronal angular velocity	−0.215	−0.078
Peak trunk sagittal angular velocity	−0.019	−0.008
Peak trunk transverse angular velocity	0.000	0.000
Peak trunk sway angle	1.397	0.686
Peak arm angular velocity	−0.001	0.000
Arm symmetry index	−0.088	−0.066
Average steering duration	0.000	0.000
Average number of steps during steering	0.997	0.542
Peak angular velocity during steering	−0.081	−0.055
Average angular velocity during steering	−0.006	0.000
Average time per step during steering	−6.749	−2.501

WBC, white blood cell; RBC, red blood cell; PLT, platelet; ALT, alanine aminotransferase; AST, aspartate aminotransferase; BG, blood glucose; LDL, low-density lipoproteins; TG, triglycerides; HDL, high-density lipoproteins; TC, total cholesterol; MoCA, montreal cognitive assessment; MMSE, mini-mental state examination.

### 3.3 Eigenvalues, variance contribution rates, and cumulative contribution rates of principal components in PCA

Principal component analysis (PCA) was performed on the variables selected from all participants. The eigenvalues of the first six principal components were *λ*1 = 5.486, *λ*2 = 2.151, *λ*3 = 1.920, *λ*4 = 1.822, *λ*5 = 1.335, and *λ*6 = 1.142, all of which exceeded 1. The corresponding variance contribution rates of the first to sixth principal components were 24.934%, 9.779%, 8.726%, 8.284%, 6.068%, and 5.191%, respectively. The cumulative variance contribution rate of the first six principal components reached 62.983%, indicating that these six components captured 62.983% of the information contained in the original 22 variables. Therefore, retaining six principal components was considered appropriate. This 6-component cutoff, strictly dictated by the Kaiser criterion (Eigenvalue > 1), optimally balances essential physiological signal retention with the rigorous filtration of non-generalizable noise. Given the substantially higher contribution rate of the first principal component compared with the others, the distributions of the first two principal components in two-dimensional space were further examined. Detailed results are presented in Table 3 and Figure 3.

**Table 3:** Eigenvalues, variance contribution rates, and cumulative contribution rates of the correlation matrix among participants.

Component	Initial eigenvalues			Extracted eigenvalues		
	*λ*	Variance contribution rate (%)	Cumulative contribution rate (%)	*λ*	Variance contribution rate (%)	Cumulative contribution rate (%)
1	5.486	24.934	24.934	5.486	24.934	24.934
2	2.151	9.779	34.713	2.151	9.779	34.713
3	1.920	8.726	43.439	1.920	8.726	43.439
4	1.822	8.284	51.723	1.822	8.284	51.723
5	1.335	6.068	57.791	1.335	6.068	57.791
6	1.142	5.191	62.983	1.142	5.191	62.983
7	0.963	4.375	67.358			
8	0.925	4.205	71.563			
9	0.876	3.980	75.543			
10	0.730	3.318	78.860			
11	0.707	3.214	82.074			
12	0.629	2.859	84.933			
13	0.560	2.547	87.480			
14	0.477	2.169	89.649			
15	0.459	2.086	91.735			
16	0.433	1.970	93.704			
17	0.337	1.532	95.237			
18	0.321	1.459	96.696			
19	0.266	1.210	97.905			
20	0.188	0.853	98.758			
21	0.171	0.777	99.535			
22	0.102	0.465	100.000			

**Figure 3:**
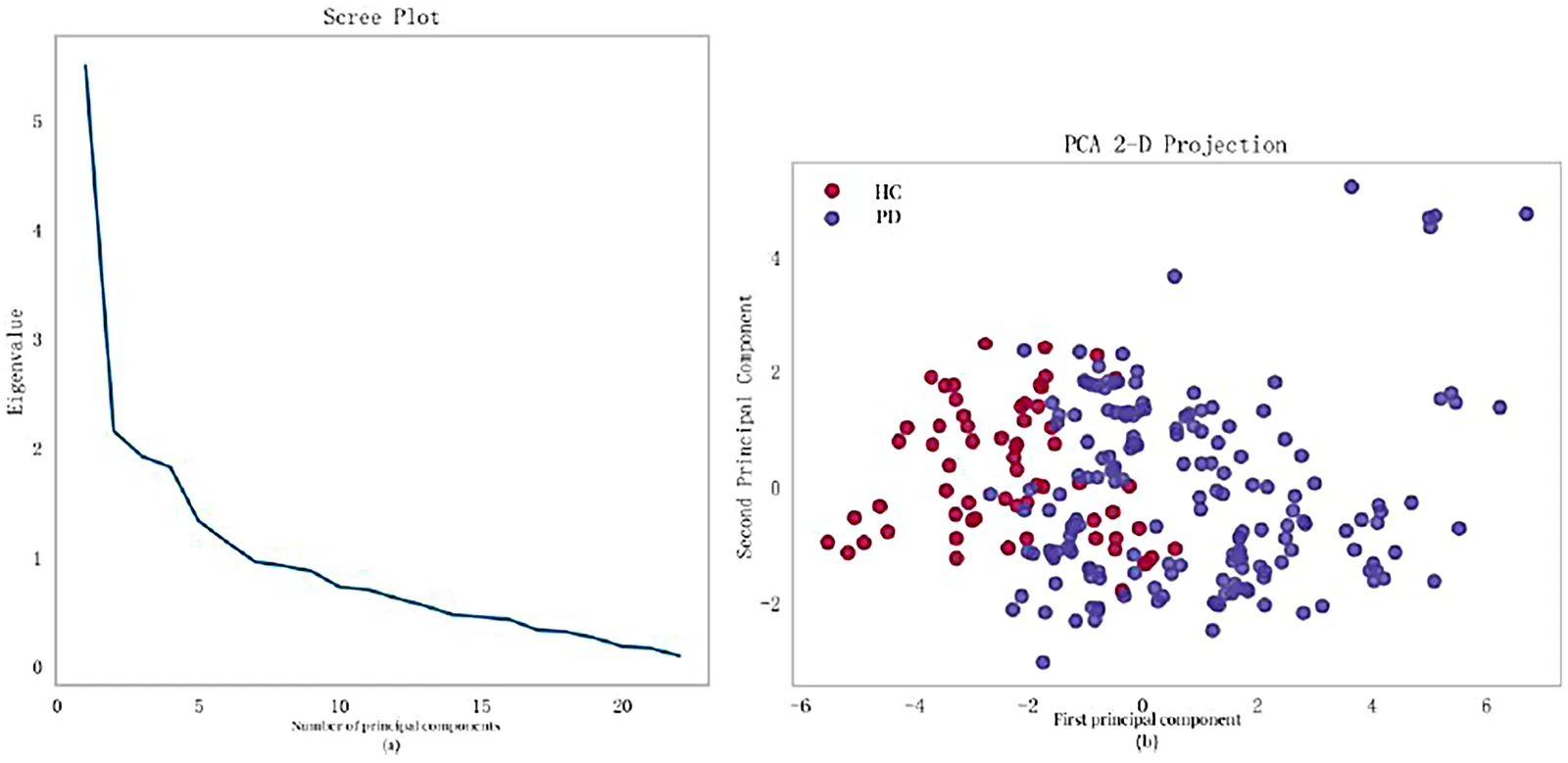
**(a)** Scree plot showing the distribution of eigenvalues for participant-related variables; **(b)** two-dimensional distribution of the first two principal components in the PD and HC groups.

### 3.4 Eigenvectors of the first six principal components

Detailed calculation results are presented in Table 4. The expression for principal component 1 was as follows: PC1 = −0.272 × WBC − 0.063 × PLT − 0.098 × ALT + … − 0.167 × average time per step during steering. The expression for principal component 2 was: PC2 = 0.086 × WBC + 0.174 × PLT + 0.203 × ALT + … + 0.066 × average time per step during steering. The expression for principal component 3 was: PC3 = 0.287 × WBC + 0.122 × PLT + 0.376 × ALT + … − 0.333 × average time per step during steering. The expression for principal component 4 was: PC4 = −0.079 × WBC + 0.402 × PLT + 0.043 × ALT + … − 0.200 × average time per step during steering. The expression for principal component 5 was: PC5 = −0.231 × WBC − 0.079 × PLT + 0.446 × ALT + … + 0.486 × average time per step during steering. The expression for principal component 6 was: PC6 = −0.007 × WBC − 0.188 × PLT − 0.054 × ALT + … + 0.063 × average time per step during steering. Clinically, based on these primary eigenvector loadings, the extracted principal components can be interpreted as follows: PC1 captures global kinematic vigor and cognitive-motor pace; PC2 reflects axial postural stability and bilateral coordination; PC3 synthesizes the systemic hepato-metabolic profile with dynamic turning velocity; PC4 represents axial trunk rigidity coupled with hematological inflammatory shifts; PC5 specifically isolates steering hesitation and turning kinematics; and PC6 reflects gait rhythmicity and interlimb phase synchronization.

**Table 4:** Eigenvector matrix of the first six principal components.

Eigenvectors	PC1	PC2	PC3	PC4	PC5	PC6
WBC	−0.272	0.086	0.287	−0.079	−0.231	−0.007
PLT	−0.063	0.174	0.122	0.402	−0.079	−0.188
ALT	−0.098	0.203	0.376	0.043	0.446	−0.054
AST	−0.184	0.037	0.381	0.018	0.232	0.054
Creatinine	−0.085	0.199	0.151	−0.301	0.015	0.278
HDL	0.245	0.124	−0.124	0.235	−0.005	0.054
TC	−0.072	0.238	0.356	0.117	0.231	0.042
MOCA	0.292	−0.017	−0.167	0.174	0.247	0.050
Stride length	0.334	−0.041	−0.108	0.030	0.224	0.142
Peak shank angular velocity	0.339	−0.022	0.115	−0.186	0.034	−0.133
Shank symmetry index	−0.038	0.379	−0.194	0.256	0.115	−0.125
Relative mean phase difference	−0.061	0.233	−0.016	0.023	−0.188	0.611
Interlimb coordination	0.015	0.389	−0.206	0.108	−0.158	0.413
Peak trunk coronal angular velocity	0.113	0.441	−0.056	−0.280	−0.102	−0.276
Peak trunk sagittal angular velocity	0.289	0.125	0.145	−0.098	−0.204	−0.228
Peak trunk sway angle	−0.072	0.350	−0.159	−0.467	0.011	−0.186
Peak arm angular velocity	0.217	0.209	0.030	0.003	−0.186	−0.118
Arm symmetry index	0.115	0.273	0.011	0.347	0.105	−0.141
Average number of steps during steering	−0.298	0.008	−0.041	0.236	−0.311	−0.134
Peak angular velocity during steering	0.315	−0.001	0.309	−0.069	−0.130	0.143
Average angular velocity during steering	0.333	−0.029	0.245	−0.022	0.010	0.195
Average time per step during steering	−0.167	0.066	−0.333	−0.200	0.486	0.063

PLT, platelet; ALT, alanine aminotransferase; AST, aspartate aminotransferase; HDL, high-density lipoproteins; TC, total cholesterol; MoCA, montreal cognitive assessment.

### 3.5 Logistic machine learning analysis and external validation

To construct a robust diagnostic model and explicitly prevent information leakage, we implemented a nested cross-validation framework followed by testing on a strictly independent external cohort (*N* = 36). The machine learning pipeline strictly confined LASSO feature selection and PCA dimensionality reduction to the training folds during each cross-validation iteration. Under this rigorous leakage-free workflow, the original-variable group achieved a mean cross-validated AUC of [0.942 (0.901–0.982)] with an accuracy of [0.823 (0.750–0.895)] on the internal validation sets. The nested PCA group achieved a mean AUC of [0.949 (0.914–0.985)] with an accuracy of [0.827 (0.769–0.886)] (Figure 4a). As detailed in Supplementary Table S1, integrating wearable gait signatures significantly elevated the “Clinical-Only” baseline AUC from 0.881 (95% CI: 0.825–0.938) to 0.949 (95% CI: 0.914–0.985), proving their additive diagnostic value (Supplementary Table S2 adjusts for Age). Figure 4 presents the performance and interpretability of Logistic regression models based on original variables and PCA-derived variables, including ROC curves, learning curves, calibration, decision curve, and SHAP analyses. Based on the AUC metric, the performance of the validation set in both groups did not exceed that of the testing set, or exceeded it by less than 10%, indicating satisfactory model fitting. Therefore, the Logistic model was considered suitable for classification modeling in this dataset (Figure 4b). The Calibration curve analysis value in the PCA group was 0.083 (95% CI, 0.058–0.108), while the value in the original data group was 0.098 (95% CI, 0.066–0.133) (Figure 4c). Decision curve analysis showed that the maximum net benefit value of PCA group was 0.738, and the maximum net benefit value of original data group was also 0.738 (Figure 4d). Figure 4e was SHAP feature importance analysis chart, showing the relative contribution degree and importance ranking of each feature to model prediction results. Supplementary Figure S1 further presents the classification results of the original-variable and PCA-based models in the training and test sets, including the numbers of correctly and incorrectly classified cases. Overall, in this exploratory study, the PCA-based logistic regression model demonstrated better diagnostic performance than the model based on the original variables.

**Figure 4:**
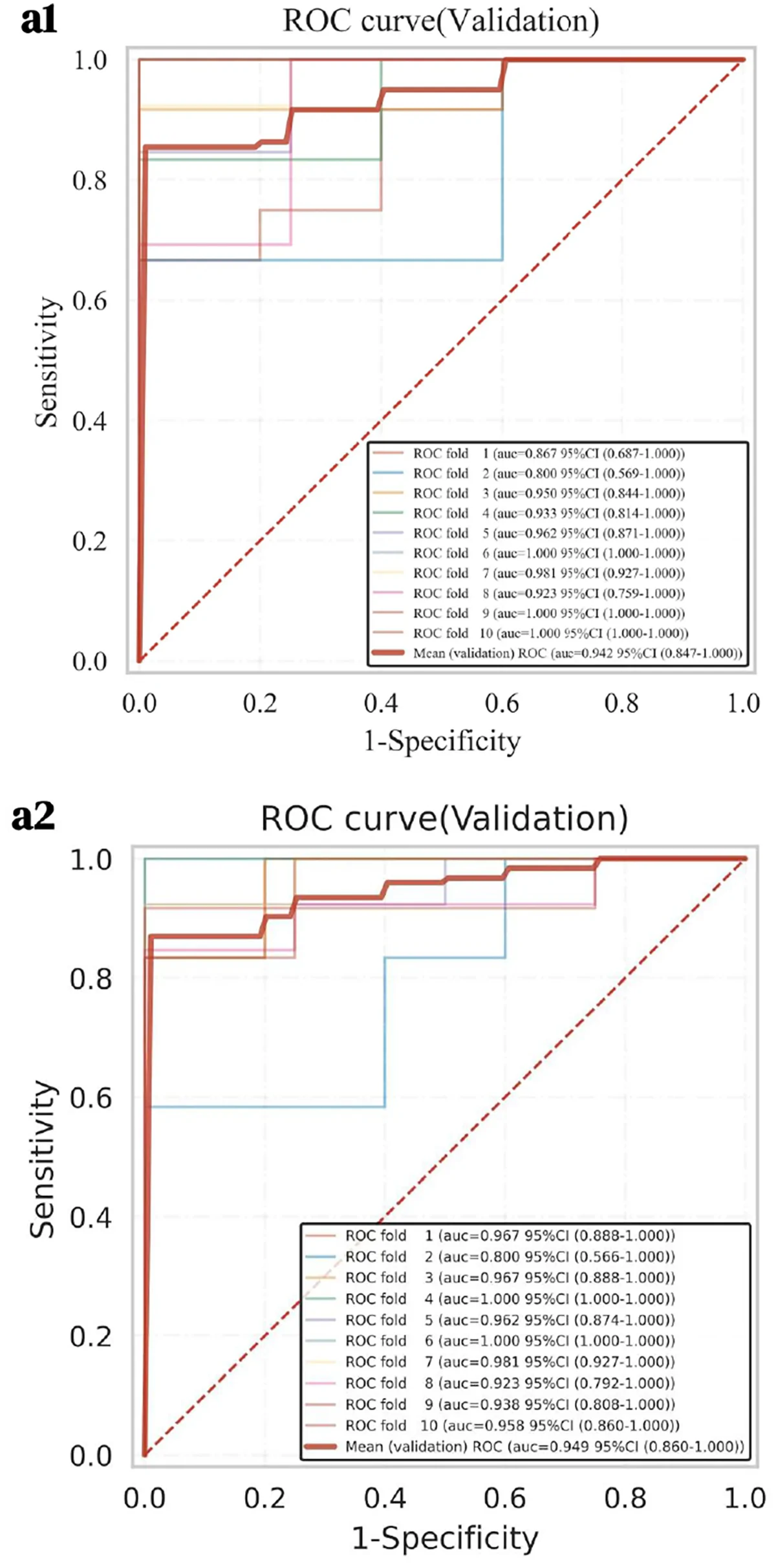
Logistic regression models. Suffix “1” indicates the original-variable group, whereas suffix “2” indicates the PCA group. **(a)** ROC curves and AUC values for the validation set. Solid lines in different colors represent the results from the 10-fold cross-validation. **(b)** Learning curves of the Logistic regression models. The red dashed line represents the training set, whereas the blue dashed line represents the validation set. **(c)** Calibration curve analysis (the closer you get to zero, the better). **(d)** Decision curve analysis. **(e)** Figure of the results of SHAP rose plot analysis.

**Figure:**
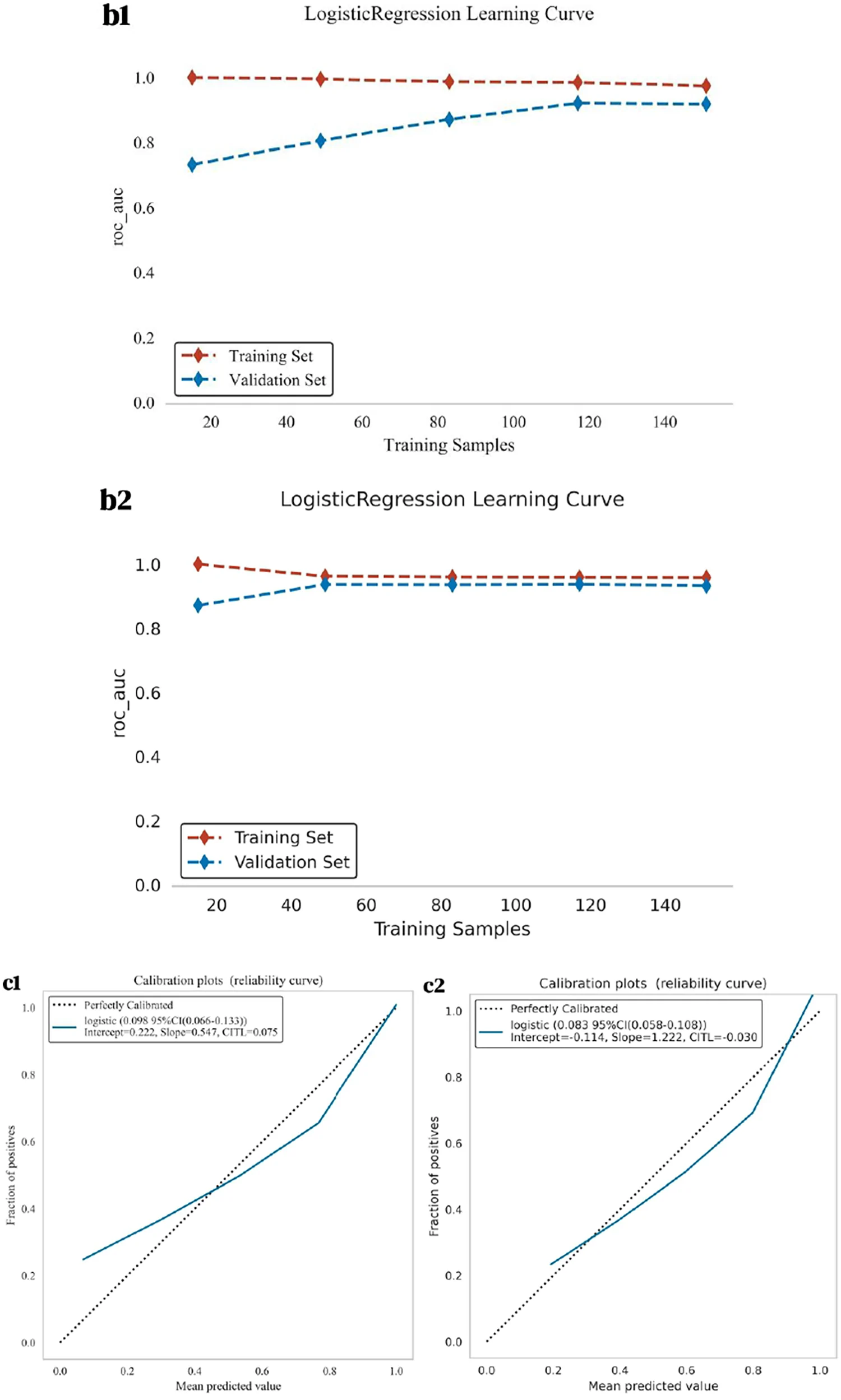


**Figure:**
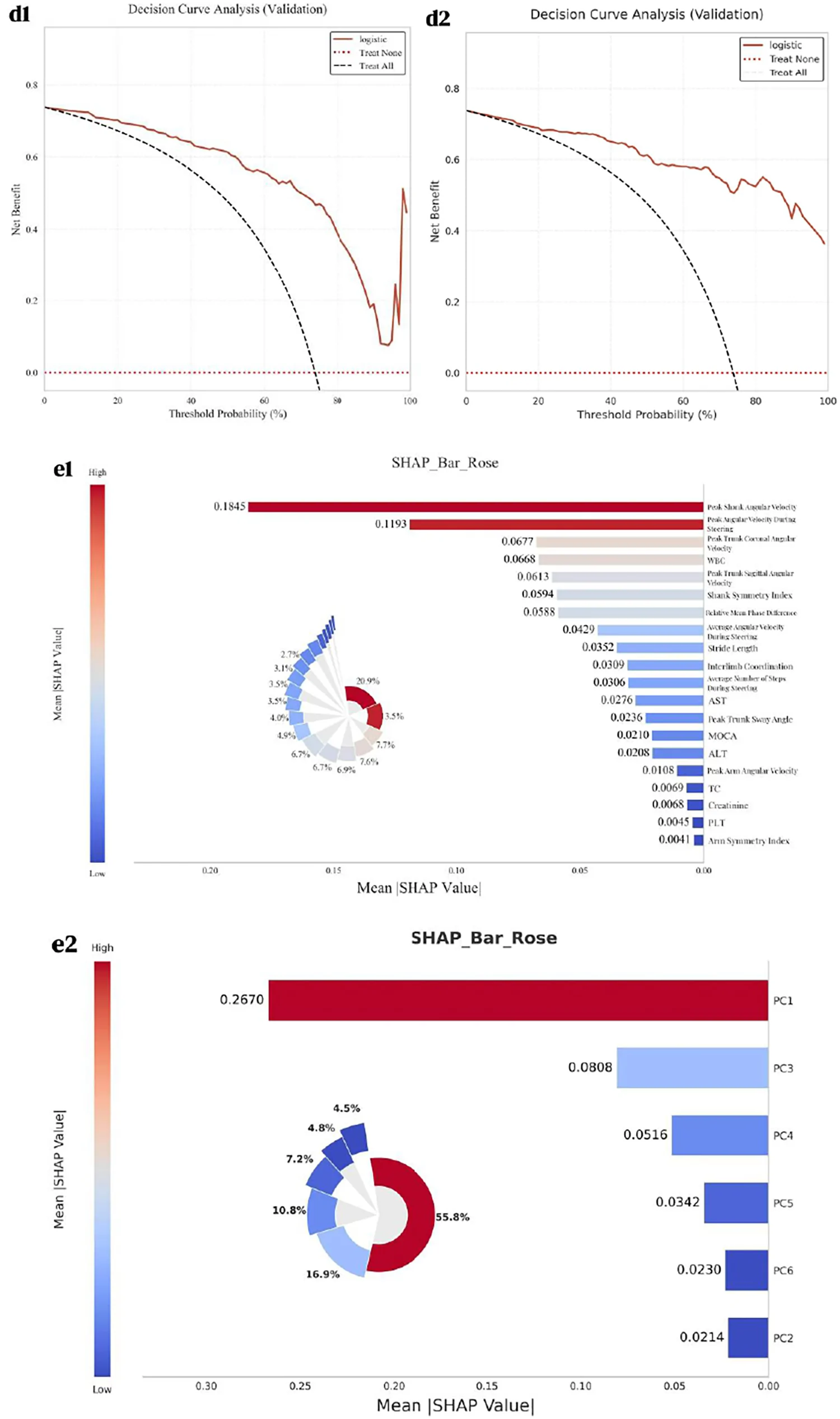


Comparative evaluation of seven classifiers showed consistently higher internal cross-validation performance when models were based on the 22 LASSO-selected variables, whereas PCA-based models demonstrated more moderate and stable performance (Supplementary Table S3). Among the PCA-based models, SVM achieved the highest AUC (0.969), followed by Logistic Regression (AUC = 0.942). Given its competitive discriminative performance, transparent parameter interpretation, and greater clinical interpretability compared with more complex classifiers, PCA-based Logistic Regression was selected as the final diagnostic framework. Furthermore, a targeted subgroup analysis isolating the predominantly early-stage PD cohort (Hoehn and Yahr stages 1–2, comprising 84.75% of the patients) confirmed that the PCA-Logistic framework maintained highly robust discriminative performance with an AUC of 0.928 (Supplementary Table S4), definitively proving that the model's diagnostic efficacy is highly sensitive to early pathological kinematics and not artificially driven by advanced disease phenotypes.

Most importantly, to definitively validate the generalizability of the proposed framework, the trained models were evaluated on the completely unseen external cohort of 36 independent subjects (who did not participate in any prior feature selection or data transformation steps). In the independent test set, the PCA-based logistic regression model demonstrated robust diagnostic performance, achieving an AUC of 0.891, an accuracy of 0.750, a sensitivity of 0.760, a specificity of 0.727, a positive predictive value of 0.864, a negative predictive value of 0.571, an *F*1 score of 0.809, and Kappa of 0.453. Figures 5a–d presents the ROC curve, calibration curve, decision curve analysis, and confusion matrix heatmap for this cohort.

**Figure 5:**
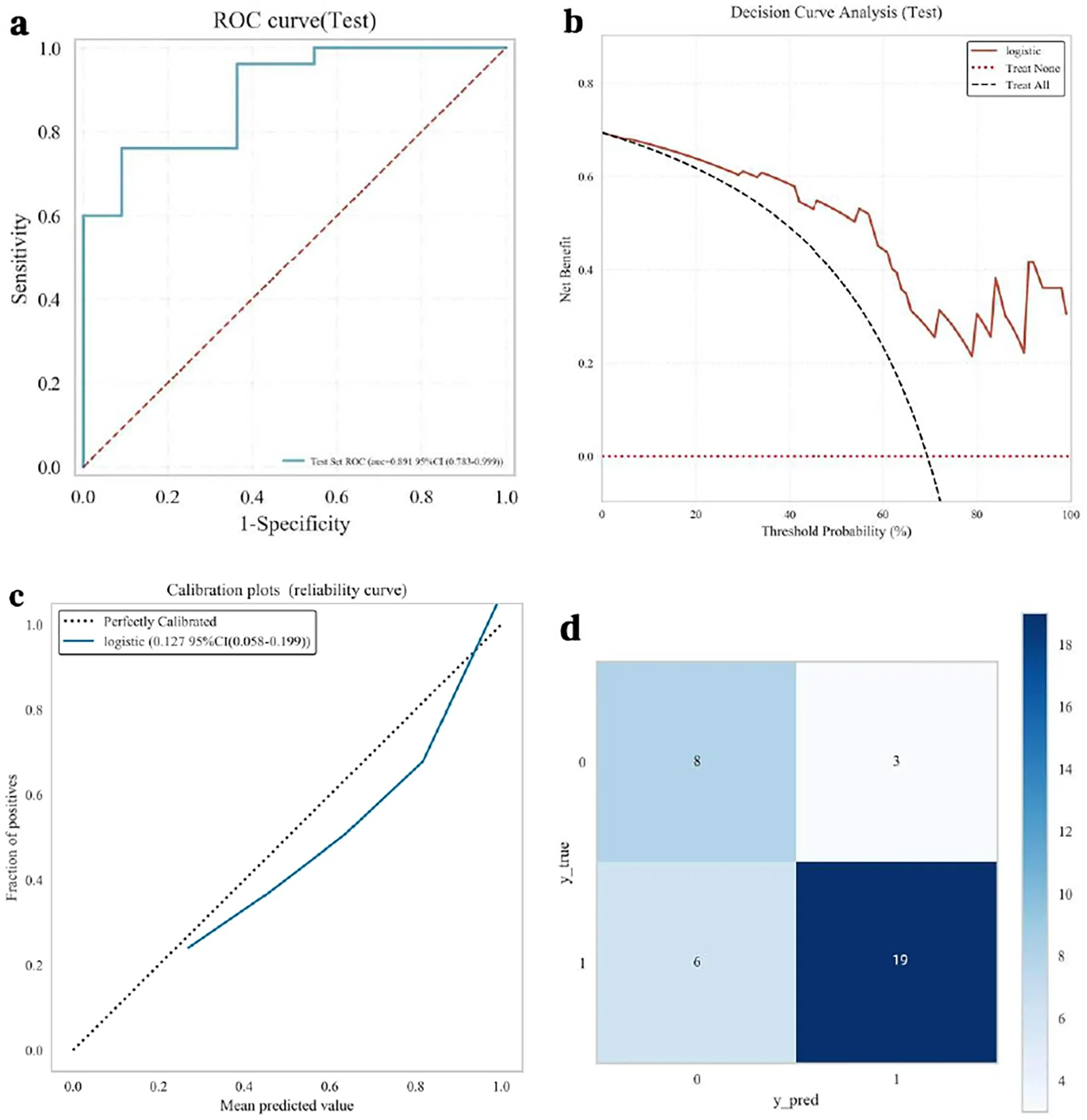
External validation cohort. **(a)** ROC curves. **(b)** Decision curve analysis (the maximum net benefit was 0.694). **(c)** Calibration curve analysis (the closer you get to zero, the better). **(d)** Confusion matrix heatmap.

## 4 Discussion

This exploratory study aimed to evaluate the diagnostic potential of wearable sensor-derived gait parameters for Parkinson's disease (PD) using principal component analysis (PCA) combined with logistic regression modeling. Baseline comparison revealed no statistically significant differences in clinical characteristics between the PD and control groups, with the exception of Age. Subsequently, Age, hematological indices, cognitive parameters, and kinematic variables were entered into a least absolute shrinkage and selection operator (LASSO) regression analysis, which identified 22 candidate predictors. PCA was then applied to reduce the dimensionality of these 22 variables, yielding 6 principal components that collectively explained 62.983% of the total variance in the original feature set, thereby providing a compact representation of the underlying risk profile. Logistic regression models were separately constructed using (i) the 22 original variables selected by LASSO and (ii) the 6 PCA-derived components. To rigorously evaluate diagnostic performance without information leakage, these models were constructed utilizing a nested cross-validation framework. The PCA-based model achieved an excellent internal mean AUC of 0.949 with an accuracy of 0.827. Most importantly, when evaluated on a completely independent, naive external validation cohort (*N* = 36), the finalized PCA-based model continued to yield a robust AUC of 0.891 with an accuracy of 0.750. These findings unequivocally demonstrate that the PCA-reduced model, built upon wearable sensor-derived gait parameters, not only exhibits strong diagnostic performance for PD but also possesses genuine generalizability across unseen datasets, successfully overcoming the overfitting limitations commonly seen in high-dimensional biological data. As an exploratory investigation, this study suggests that the integration of wearable sensor-derived gait parameters with machine learning approaches holds considerable potential for clinical deployment in primary care settings, offering a theoretical basis and practical reference for the early screening and auxiliary diagnosis of PD in community-level healthcare facilities. Crucially, addressing common methodological pitfalls in high-dimensional medical data analysis, this study implemented a strictly leakage-free nested machine learning workflow. By confining LASSO and PCA exclusively within the training folds and subsequently validating the final model on a completely independent cohort of 36 unseen individuals, we confirmed that the strong predictive performance of the PCA-derived components is genuinely robust and not a byproduct of information leakage or algorithm overfitting. Furthermore, a common critique of feature selection techniques like LASSO in moderate sample sizes is the uncertainty and potential instability of the selected features. The sustained high diagnostic accuracy (AUC = 0.891) achieved on this independent 36-patient cohort serves as an empirical robustness check. It demonstrates that the 22 retained features are not sample-specific noise (overfitting), but rather stable, universally applicable signatures of Parkinson's disease, thereby effectively addressing concerns related to small-sample variance. Crucially, integrating wearable gait signatures elevated the internal cross-validated AUC from 0.881 (clinical baseline alone) to 0.949. This demonstrates that wearable sensors capture unique subclinical motor anomalies missed by conventional assessments, confirming their indispensable additive diagnostic value.

Parkinson's disease (PD) is a progressive neurodegenerative disorder characterized by cardinal motor features, including tremor, rigidity, bradykinesia, and postural instability (11). Among these manifestations, gait disturbances play a particularly pivotal role in disease progression and patient prognosis (12). PD represents the fastest-growing movement disorder worldwide, currently affecting more than 6.2 million individuals globally (13). The pathophysiological basis of PD is predominantly attributed to the degeneration of dopaminergic neurons in the substantia nigra pars compacta, with profound depletion of striatal dopamine representing the primary neurochemical deficit underlying the disease (14, 15). The substantia nigra plays a critical role in motor coordination; its degeneration disrupts the dynamic equilibrium between excitatory and inhibitory signaling within basal ganglia circuits, thereby giving rise to the hallmark symptoms of PD (16). These motor symptoms arise directly from this circuit-level dysfunction, producing characteristic movement impairments that substantially compromise patients' functional mobility. In early-stage PD, gait disturbances are often subtle and easily overlooked by patients and their families; however, the underlying neuropathological processes may have already been initiated and are progressively advancing, with ongoing deterioration of the dopaminergic system. The brain possesses robust compensatory mechanisms—including upregulation of activity in unaffected brain regions and increased sensitivity of dopamine receptors—that enable most patients with early PD to maintain apparently normal motor function during routine daily activities (17). As PD advances, gait impairments become increasingly prominent, ultimately leading to recurrent falls, progressive disability, and marked deterioration in quality of life (18). Therefore, quantitative gait assessment is essential for early diagnosis and longitudinal disease monitoring. In particular, the capacity to detect subtle yet clinically meaningful changes in gait under real-world conditions may provide valuable insights into the efficacy of emerging therapeutic interventions for PD. Advances in technology have established wearable sensor systems as a mature and validated measurement platform capable of providing continuous, real-time gait data in free-living environments (19). This technology thus provides the objective and reliable technical foundation upon which the present study was designed.

Wearable sensors have emerged as a powerful tool for gait parameter quantification, offering a non-intrusive, scalable, and non-invasive approach with the advantages of convenience and the capacity for continuous monitoring (20, 21). Wearable sensors—particularly accelerometers and gyroscopes—have introduced innovative approaches to gait analysis by providing real-time, multi-axis motion data (22–24). Accelerometers detect linear acceleration along the *X*, *Y*, and *Z* axes, whereas gyroscopes measure rotational motion, enabling the capture of subtle movements and irregularities within gait patterns (23). These devices can be worn comfortably by patients, facilitating unobtrusive, long-term monitoring in naturalistic daily-life environments. This advantage renders wearable sensors ideally suited for tracking PD progression over time, providing a continuous data stream without the need for frequent hospital visits or specialized laboratory equipment (25–27). Unlike conventional gait assessment methods—such as clinical observation or video-based motion analysis—wearable sensors can objectively quantify kinematic parameters and detect subtle gait changes, revealing early motor manifestations that may otherwise remain unrecognized. These devices enable continuous collection of gait data—including step length, cadence, walk speed, symmetry, and variability—which can be analyzed to identify deviations from normative movement patterns associated with PD (25, 28, 29). Such capabilities have contributed to substantial advances in the understanding and clinical management of PD, as they allow healthcare providers to longitudinally monitor patients' motor status in their natural living environments. Numerous studies have validated accelerometer-based systems for measuring gait abnormalities in PD. An early study by Liu et al. provided key evidence for the reliability and validity of accelerometry-based gait assessment (30). Their findings demonstrated that wearable sensors can capture subtle gait abnormalities—such as fluctuations in cadence and irregularities in stride length—that are typically difficult to detect using conventional clinical methods, thereby laying the groundwork for the application of accelerometry in both research and clinical settings (30). Subsequent studies in the PD field have further demonstrated that accelerometer-based wearable monitors can reliably discriminate between patients with PD and healthy controls (31, 32). For instance, Silvia et al. found that accelerometers could detect significant differences in gait patterns between PD patients and controls, including reduced walk speed, shorter step length, and diminished gait symmetry—all recognized as motor hallmarks of PD (32). Similarly, Yoav et al. demonstrated that inertial sensor-derived gait parameters exhibited favorable diagnostic performance in distinguishing PD patients from healthy individuals (31). These findings are consistent with the results of the present study, in which wearable sensor-derived gait parameters effectively discriminated between PD patients and healthy controls. Notably, the wearable system employed in this study incorporated a greater number of sensors with higher precision compared with those used in previous investigations. Furthermore, this study highlights the capacity of wearable sensors to detect temporal changes in gait, which is critical for monitoring disease progression and evaluating therapeutic efficacy. The continuous data provided by wearable sensors may facilitate more accurate and timely adjustments to treatment regimens, ultimately improving patient outcomes and optimizing clinical decision-making.

Despite the promise of wearable sensors in gait assessment, the challenge of extracting meaningful information from the vast volume of data collected remains substantial. Dimensionality reduction techniques—such as LASSO regression and principal component analysis (PCA)—have emerged as key tools for addressing this challenge. LASSO regression can analyze complex datasets while retaining the most informative features and minimizing overfitting (33). By applying L1-penalized logistic regression, LASSO effectively eliminates features with negligible predictive value, thereby enhancing model interpretability, generalizability, and accuracy (34). By identifying the features most sensitive to disease progression, LASSO regression supports longitudinal monitoring. Moreover, feature variables selected through LASSO have been shown to effectively discriminate between patients and healthy controls (35, 36). By selecting the subset of variables that most effectively capture disease-specific characteristics, LASSO can generate parsimonious predictive models without compromising accuracy. This rationale underpins the selection of LASSO as the primary feature selection method in the present study. Clinical review of the LASSO-selected features showed that the retained variables were broadly consistent with clinically interpretable measures, including MoCA and turning-related gait parameters. Subsequent PCA further integrated correlated features into orthogonal components, reducing dependence on arbitrary single-feature selection while preserving clinically relevant information. Biologically, the retention of specific routine laboratory biomarkers by the LASSO algorithm aligns rigorously with the pathophysiology of PD; specifically, markers such as WBC and PLT capture the systemic peripheral-central neuroinflammatory cross-talk, while HDL and TC reflect the underlying lipid metabolism dysregulation inextricably linked to neurodegeneration. Following initial feature selection via LASSO, this study additionally incorporated PCA as a complementary dimensionality reduction technique. PCA enhances the analytical utility of wearable sensor data by simplifying complex datasets while preserving the most informative content. By transforming original gait features into a reduced set of uncorrelated components, PCA can identify the movement patterns most predictive of PD. Wearable sensors generate multidimensional datasets that capture subtle variations in gait dynamics, including step length, walk speed, stride length, cadence, symmetry, and stride variability. Although these parameters are advantageous for identifying movement disorders, the large number of features commonly introduces challenges such as multicollinearity and overfitting in both statistical and machine learning models. Without appropriate data preprocessing, models may struggle to identify the most predictive features, potentially compromising their accuracy and reliability. Furthermore, high-dimensional datasets are computationally intensive, requiring advanced techniques to distill meaningful patterns. This is particularly relevant in clinical settings, where the objective is to develop interpretable models that can inform diagnostic and therapeutic decision-making. PCA offers a solution by reducing data complexity and highlighting the most informative components (37). PCA has been applied across a range of studies to extract meaningful insights from gait data. For example, Matsushima et al. employed PCA to analyze data collected from triaxial accelerometers, revealing the principal components underlying ataxic gait (38). This study underscored PCA's ability to identify patterns characteristic of movement disorders—such as reduced walk speed, shorter step length, and diminished regularity and symmetry—that were less pronounced in control subjects (38). Nguyen et al. further explored the application of PCA to data from small, wireless wearable gait sensors, demonstrating that principal components can capture gait variations closely associated with individual fall risk (39). In a large-scale population-based cohort study, PCA applied to 30 gait parameters yielded seven independent gait domains that effectively summarized distinct dimensions of walking performance (40). In the present study, PCA was applied to the 22 original parameters identified through LASSO regression, yielding six principal components. These six components collectively captured 62.983% of the total variance in the original 22 features, providing a compact representation of the overall risk profile and establishing a foundation for machine learning-based classification. By integrating PCA with machine learning techniques, the robustness and accuracy of predictive models can be enhanced, highlighting the potential of this combined approach for PD diagnosis and disease monitoring. Across the evaluated classifiers, PCA-based models showed more stable internal performance than models using the 22 LASSO-selected variables directly. Although SVM achieved slightly higher discrimination than Logistic Regression, PCA-based Logistic Regression was selected as the final model because of its competitive performance and greater interpretability for clinical application. Furthermore, an age-adjusted sensitivity analysis confirmed that the PCA-Logistic framework maintained highly robust discriminative capacity (AUC = 0.950, 95% CI: 0.916–0.985), demonstrating that the integrated digital and biological signatures are genuinely disease-specific and resilient to demographic confounding (Supplementary Table S2).

By reducing data complexity, PCA facilitates the development of interpretable models that can be readily implemented in clinical settings. However, one of the most promising applications of PCA lies in its integration with machine learning algorithms (41). By preprocessing gait data with PCA, researchers can reduce noise and redundancy, thereby streamlining the inputs to machine learning models. This preprocessing step not only improves computational efficiency but also enhances model performance by focusing on the most relevant features (42). In a study by Zheng et al., PCA was used to preprocess multidimensional CT imaging data prior to applying a support vector machine classifier; the combined approach substantially improved overall model accuracy (43). Similarly, Grunert et al. combined PCA with supervised machine learning models to achieve high classification accuracy, demonstrating the effectiveness of this approach for binary classification tasks (44). In the present study, integrating PCA with logistic regression mitigated the challenges posed by high-dimensional data while simultaneously enhancing the performance and interpretability of the classification model. Ivanov et al. investigated the application of PCA combined with multiple machine learning algorithms for constructing cancer assessment models with robust predictive features (45). Their findings demonstrated that PCA effectively reduced redundancy within the dataset by focusing on the most informative features while maintaining diagnostic accuracy; when combined with logistic regression, the model exhibited higher sensitivity and specificity, underscoring the value of integrating these approaches in clinical applications (45). This evidence provided a key rationale for the selection of logistic regression as the classification algorithm in the present study. In this study, logistic regression models were separately constructed using the 22 original parameters selected by LASSO and the six PCA-derived principal components. The results indicated that the PCA-based model demonstrated superior diagnostic performance for PD. This finding further supports the clinical applicability of this combined approach. Dimensionality reduction techniques such as LASSO and PCA play a critical role in maximizing the utility of wearable sensor data for PD diagnosis and monitoring. By simplifying complex datasets and isolating the most relevant features, PCA enhances both the performance and interpretability of classification models. Its integration with machine learning algorithms further underscores its potential to advance PD research and clinical practice. As wearable sensor technology continues to evolve, PCA is likely to remain a cornerstone of clinical data analysis, driving innovation in PD diagnosis and management. Crucially, to explicitly counter the abstraction inherently introduced by PCA across heterogeneous data types, we integrated SHAP (SHapley Additive exPlanations) value analyses (Figure 4e); by coupling the SHAP global feature importance with the PCA eigenvector matrix (Table 4), clinicians can transparently trace the model's predictive logic backward from the abstract principal components to the precise, original serological and kinematic scales, thereby ensuring uncompromised clinical interpretability.

In summary, the integration of wearable gait sensor data with LASSO–PCA dimensionality reduction and logistic regression modeling provides a robust and interpretable framework for PD classification, in which PCA-compressed features demonstrated particular strengths in diagnostic efficiency and model generalizability. This approach enables real-time, objective quantification of gait abnormalities, offering clinicians a more comprehensive tool for motor function assessment and providing a scalable, non-invasive pathway for early screening, accurate diagnosis, and longitudinal disease monitoring. As the underlying technologies continue to advance, this sensor-driven analytical paradigm has the potential to become an integral component of individualized PD care; however, further validation in multicenter, longitudinal cohorts is warranted to establish its clinical applicability and external generalizability.

This study has several limitations that should be acknowledged. First, while we successfully validated the model on an independent external cohort, the overall sample size remains relatively small; future studies with larger cohorts enrolled across multiple centers and with longitudinal follow-up are needed to further validate the clinical applicability of our findings. Second, gait patterns in patients with PD are inherently variable, which complicates the interpretation of study results. Environmental factors—such as footwear type, walking surface, and fatigue status—may also have influenced sensor readings during data collection. Future studies should implement standardized testing conditions and enroll larger, more diverse populations to enhance the stability and generalizability of the findings. Third, although evaluating patients exclusively in the “ON” state potentially masked macroscopic deficits, the algorithm successfully captured levodopa-resistant axial signatures (e.g., turning kinematics). Nonetheless, future validation in treatment-naive or “OFF” state cohorts is strictly required to establish its unconfounded diagnostic efficacy. Furthermore, the linear assumptions inherent to PCA and logistic regression may oversimplify the complex dynamics of Parkinsonian gait. Future large-scale studies must transcend these limitations by adopting advanced non-linear machine learning architectures and integrating non-linear signal processing techniques, such as multiscale entropy (MSE). As recent seminal studies have demonstrated, evaluating the complexity and non-linear adaptability of continuous wearable sensor-derived gait signals holds profound potential for uncovering hidden, early-stage physiological signatures of PD (46–48).

## Data Availability

The data analyzed in this study is subject to the following licenses/restrictions: the datasets generated and analyzed during the current study are not publicly available due to patient privacy and ethical restrictions. However, the original contributions presented in the study are included in the article. Further inquiries and reasonable requests to access the datasets can be directed to the corresponding authors. Requests to access these datasets should be directed to Guo Hong, E-mail address: 364939259@qq.com.

## References

[1] Mirelman A Bonato P Camicioli R Ellis TD Giladi N Hamilton JL. Gait impairments in Parkinson’s disease. Lancet Neurol. (2019) 18(7):697–708. 10.1016/S1474-4422(19)30044-4 30975519

[2] Bloem BR Okun MS Klein C. Parkinson’s disease. Lancet. (2021) 397(10291):2284–303. 10.1016/S0140-6736(21)00218-X 33848468

[3] Belić M Bobić V Badža M Šolaja N Đurić-Jovičić M Kostić VS. Artificial intelligence for assisting diagnostics and assessment of Parkinson’s disease-a review. Clin Neurol Neurosurg. (2019) 184:105442. 10.1016/j.clineuro.2019.105442 31351213

[4] Warmerdam E Hausdorff JM Atrsaei A Zhou Y Mirelman A Aminian K. Long-term unsupervised mobility assessment in movement disorders. Lancet Neurol. (2020) 19(5):462–70. 10.1016/S1474-4422(19)30397-7 32059811

[5] di Biase L Pecoraro PM Pecoraro G Shah SA Di Lazzaro V. Machine learning and wearable sensors for automated Parkinson’s disease diagnosis aid: a systematic review. J Neurol. (2024) 271(10):6452–70. 10.1007/s00415-024-12611-x 39143345

[6] Mei J Desrosiers C Frasnelli J. Machine learning for the diagnosis of Parkinson’s disease: a review of literature. Front Aging Neurosci. (2021) 13:633752. 10.3389/fnagi.2021.633752 34025389 PMC8134676

[7] Tian Y Zhang Y. A comprehensive survey on regularization strategies in machine learning. Inf Fus. (2021) 80:146–66. 10.1016/j.inffus.2021.11.005

[8] Moitra P Chaichi A Abid Hasan SM Dighe K Alafeef M Prasad A. Probing the mutation independent interaction of DNA probes with SARS-CoV-2 variants through a combination of surface-enhanced Raman scattering and machine learning. Biosens Bioelectron. (2022) 208:114200. 10.1016/j.bios.2022.114200 35367703 PMC8938299

[9] Postuma RB Berg D Stern M Poewe W Olanow CW Oertel W. MDS clinical diagnostic criteria for Parkinson’s disease. Mov Disord. (2015) 30(12):1591–601. 10.1002/mds.26424 26474316

[10] GYENNO Technologies CO. Ltd. GYENNO MATRIX-wearable motion and gait quantitative evaluation system (2022) Available online at: https://www.gyenno.com/matrix-en[EB/OL], https:// www.gyenno.com/matrix-en (Accessed July 10, 2026).

[11] Stocchi F Bravi D Emmi A Antonini A. Parkinson disease therapy: current strategies and future research priorities. Nat Rev Neurol. (2024) 20:695–707. 10.1038/s41582-024-01034-x 39496848

[12] Matar E Tinazzi M Edwards MJ Bhatia KP. Functional movement disorder as a prodromal symptom of Parkinson’s disease-clinical and pathophysiological insights. Mov Disord. (2024) 39(11):1952–9. 10.1002/mds.29958 39119738

[13] Dorsey ER Bloem BR. The Parkinson pandemic-a call to action. JAMA Neurol. (2018) 75(1):9–10. 10.1001/jamaneurol.2017.3299 29131880

[14] Berg D Crotty GF Keavney JL Schwarzschild MA Simuni T Tanner C. Path to Parkinson disease prevention: conclusion and outlook. Neurology. (2022) 99:76–83. 10.1212/WNL.0000000000200793 35970586

[15] Chitre NM Wood BJ Ray A Moniri NH Murnane KS. Docosahexaenoic acid protects motor function and increases dopamine synthesis in a rat model of Parkinson’s disease via mechanisms associated with increased protein kinase activity in the striatum. Neuropharmacology. (2020) 167:107976. 10.1016/j.neuropharm.2020.107976 32001239 PMC7110909

[16] Subramaniam SR Magen I Bove N Zhu C Lemesre V Dutta G. Chronic nicotine improves cognitive and social impairment in mice overexpressing wild type α-synuclein. Neurobiol Dis. (2018) 117:170–80. 10.1016/j.nbd.2018.05.018 29859873 PMC6051902

[17] van Nuenen BFL Weiss MM Bloem BR Reetz K Van Eimeren T Lohmann K. Heterozygous carriers of a Parkin or PINK1 mutation share a common functional endophenotype. Neurology. (2009) 72(12):1041–7. 10.1212/01.wnl.0000338699.56379.11 19038850 PMC2821837

[18] Corrà MF Vila-Chã N Sardoeira A Hansen C Sousa AP Reis I. Peripheral neuropathy in Parkinson’s disease: prevalence and functional impact on gait and balance. Brain. (2023) 146(1):225–36. 10.1093/brain/awac026 PMC9825570 35088837

[19] Sempionatto JR Nakagawa T Pavinatto A Mensah ST Imani S Mercier P. Eyeglasses based wireless electrolyte and metabolite sensor platform. Lab Chip. (2017) 17(10):1834–42. 10.1039/c7lc00192d 28470263 PMC5507201

[20] Wang X Chen LL Zhou H Xu Y Zhang H Yang W. Enriched rehabilitation improves gait disorder and cognitive function in Parkinson’s disease: a randomized clinical trial. Front Neurosci. (2021) 15:733311. 10.3389/fnins.2021.733311 34924926 PMC8674725

[21] Palumbo AV Li Z Yang EH. Trends on carbon nanotube-based flexible and wearable sensors via electrochemical and mechanical stimuli: a review. IEEE Sens J. (2022) 22(21):20102–25. 10.1109/jsen.2022.3198847

[22] Reenalda J Maartens E Homan L Buurke JHJ. Continuous three dimensional analysis of running mechanics during a marathon by means of inertial magnetic measurement units to objectify changes in running mechanics. J Biomech. (2016) 49(14):3362–7. 10.1016/j.jbiomech.2016.08.032 27616268

[23] Chambers R Gabbett TJ Cole MH Beard A. The use of wearable microsensors to quantify sport-specific movements. Sports Med. (2015) 45(7):1065–81. 10.1007/s40279-015-0332-9 25834998

[24] Nweke HF Teh YW Mujtaba G Al-garadi MA. Data fusion and multiple classifier systems for human activity detection and health monitoring: review and open research directions. Inf Fus. (2019) 46:147–70. 10.1016/j.inffus.2018.06.002

[25] Del Din S Elshehabi M Galna B Hobert MA Warmerdam E Suenkel U. Gait analysis with wearables predicts conversion to Parkinson disease. Ann Neurol. (2019) 86(3):357–67. 10.1002/ana.25548 31294853 PMC6899833

[26] Pham TD Wårdell K Eklund A Salerud G. Classification of short time series in early Parkinsons disease with deep learning of fuzzy recurrence plots. IEEE/CAA J Autom Sin. (2019) 6(6):1306–17. 10.1109/jas.2019.1911774

[27] van Wamelen DJ Hota S Podlewska A Leta V Trivedi D Rizos A. Non-motor correlates of wrist-worn wearable sensor use in Parkinson’s disease: an exploratory analysis. NPJ Parkinsons Dis. (2019) 5:22. 10.1038/s41531-019-0094-4 31602393 PMC6775049

[28] Hupfeld KE Geraghty JM McGregor HR Hass CJ Pasternak O Seidler RD. Differential relationships between brain structure and dual task walking in young and older adults. Front Aging Neurosci. (2022) 14:809281. 10.3389/fnagi.2022.809281 35360214 PMC8963788

[29] Liu C-L Cheng F-Y Wei M-J Liao Y-Y. Effects of exergaming-based tai chi on cognitive function and dual-task gait performance in older adults with mild cognitive impairment: a randomized control trial. Front Aging Neurosci. (2022) 14:761053. 10.3389/fnagi.2022.761053 35370622 PMC8965318

[30] Liu T Inoue Y Shibata K. Development of a wearable sensor system for quantitative gait analysis. Measurement. (2009) 42(7):978–88. 10.1016/j.measurement.2009.02.002

[31] Beck Y Herman T Brozgol M Giladi N Mirelman A Hausdorff JM. SPARC: a new approach to quantifying gait smoothness in patients with Parkinson’s disease. J Neuroeng Rehabil. (2018) 15(1):49. 10.1186/s12984-018-0398-3 29914518 PMC6006701

[32] Del Din S Godfrey A Galna B Lord S Rochester L. Free-living gait characteristics in ageing and Parkinson’s disease: impact of environment and ambulatory bout length. J Neuroeng Rehabil. (2016) 13(1):46. 10.1186/s12984-016-0154-5 27175731 PMC4866360

[33] Liang W Liang H Ou L Chen B Chen A Li C. Development and validation of a clinical risk score to predict the occurrence of critical illness in hospitalized patients with COVID-19. JAMA Intern Med. (2020) 180(8):1081–9. 10.1001/jamainternmed.2020.2033 32396163 PMC7218676

[34] Sauerbrei W Royston P Binder H. Selection of important variables and determination of functional form for continuous predictors in multivariable model building. Stat Med. (2007) 26(30):5512–28. 10.1002/sim.3148 18058845

[35] Wu M-J Mwangi B Bauer IE Passos IC Sanches M Zunta-Soares GB. Identification and individualized prediction of clinical phenotypes in bipolar disorders using neurocognitive data, neuroimaging scans and machine learning. Neuroimage. (2017) 145(Pt B):254–64. 10.1016/j.neuroimage.2016.02.016 26883067 PMC4983269

[36] Tang C Fang M Tan G Zhang S Yang B Li Y. Discovery of novel circulating immune complexes in lupus nephritis using immunoproteomics. Front Immunol. (2022) 13:850015. 10.3389/fimmu.2022.850015 35419005 PMC8996714

[37] Scharf F Widmann A Bonmassar C Wetzel N. A tutorial on the use of temporal principal component analysis in developmental ERP research - opportunities and challenges. Dev Cogn Neurosci. (2022) 54:101072. 10.1016/j.dcn.2022.101072 35123341 PMC8819392

[38] Matsushima A Yoshida K Genno H Ikeda SI. Principal component analysis for ataxic gait using a triaxial accelerometer. J Neuroeng Rehabil. (2017) 14(1):37. 10.1186/s12984-017-0249-7 28464831 PMC5414235

[39] Nguyen TQ Young JH Rodriguez A Zupancic S Lie DYC. Differentiation of patients with balance insufficiency (vestibular hypofunction) versus normal subjects using a low-cost small wireless wearable gait sensor. Biosensors. (2019) 9(1):29. 10.3390/bios9010029 30813585 PMC6468670

[40] Adams HHH Verlinden VJA Callisaya ML Van Duijn CM Hofman A Thomson R. Heritability and genome-wide association analyses of human gait suggest contribution of common variants. J Gerontol A Biol Sci Med Sci. (2016) 71(6):740–6. 10.1093/gerona/glv081 26219847 PMC4888380

[41] Marukatat S. Tutorial on PCA and approximate PCA and approximate kernel PCA. Artif Intell Rev. (2022) 56(6):5445–77. 10.1007/s10462-022-10297-z

[42] Perez-Rathke A Fahie MA Chisholm C Liang J Chen M. Mechanism of OmpG pH-dependent gating from loop ensemble and single channel studies. J Am Chem Soc. (2018) 140(3):1105–15. 10.1021/jacs.7b11979 29262680 PMC5831328

[43] Zheng F Hu W Wang R. Application of hyperspectral CT technology combined with machine learning in recognition of plastic components. NDT E Int. (2019) 102:287–94. 10.1016/j.ndteint.2019.01.001

[44] Grunert T Herzog R Wiesenhofer FM Vychytil A Ehling-Schulz M Kratochwill K. Vibrational spectroscopy of peritoneal dialysis effluent for rapid assessment of patient characteristics. Biomolecules. (2020) 10(6):965. 10.3390/biom10060965 32604921 PMC7357123

[45] Ivanov D Dremin V Genova T Bykov A Novikova T Ossikovski R. Polarization-based histopathology classification of ex vivo colon samples supported by machine learning. Front Phys. (2022) 9:814787. 10.3389/fphy.2021.814787

[46] Liu Z Su D Ma L Chen H Fang J Ma H. The altered multiscale dynamics of spontaneous brain activity in depression with Parkinson’s disease. Neurol Sci. (2022) 43(7):4211–9. 10.1007/s10072-022-05974-4 35237895 PMC9213374

[47] Chung C-C Kang J-H Yuan R-Y Wu D Chen C-C Chi N-F. Multiscale entropy analysis of electroencephalography during sleep in patients with Parkinson disease. Clin EEG Neurosci. (2013) 44(3):221–6. 10.1177/1550059412475066 23545244

[48] Amisola LRV Acosta RJB Arao-Arao HMD Benitez VNC Chan RMT Co AKG. Gait analysis for Parkinson’s disease using multiscale entropy. Neurodegener Dis Manag. (2025) 15(5):245–58. 10.1080/17582024.2025.2510842 40415583 PMC12456205

