# Supplementary material for: Wearable sensor–derived gait signatures for Parkinson's disease: an exploratory diagnostic study integrating dimensionality reduction and machine learning
Source: Front Med (Lausanne). 2026 Oct 8;13:1939598. doi: 10.3389/fmed.2026.1939598 (PMC13686899; doi:10.3389/fmed.2026.1939598)
Supplement: Supplementary file 1 [file Supplementaryfile1.docx]

**Supplementary Table S1. Logistic Machine Learning Analysis related parameters of the Clinical-Only Baseline group, original variable group and the PCA group**

|  | **AUC** | **cutoff** | **Accuracy** | **Sensitivity** | **specificity** | **PPV** | **NPV** | **F1 score** | **Kappa** |
| --- | --- | --- | --- | --- | --- | --- | --- | --- | --- |
| Clinical-Only | 0.881(0.825-0.938) | 0.612(0.588-0.637) | 0.839(0.779-0.900) | 0.871(0.817-0.925) | 0.760(0.629-0.891) | 0.909(0.859-0.959) | 0.694(0.572-0.816) | 0.888(0.845-0.931) | 0.604(0.457-0.750) |
| Original | 0.942(0.901-0.982) | 0.683(0.613-0.752) | 0.823(0.750-0.895) | 0.839(0.770-0.908) | 0.795(0.640-0.950) | 0.918(0.854-0.981) | 0.653(0.555-0.750) | 0.872(0.816-0.927) | 0.582(0.421-0.742) |
| PCA | 0.949(0.914-0.985) | 0.742(0.712-0.773) | 0.827(0.769-0.886) | 0.806(0.724-0.889) | 0.895(0.804-0.986) | 0.956(0.919-0.993) | 0.660(0.551-0.769) | 0.868(0.817-0.919) | 0.620(0.514-0.727) |

**Supplementary Table S2. Logistic Machine Learning Analysis related parameters of the original variable group and the PCA group(Age was used as a covariate).**

|  | **AUC** | **cutoff** | **Accuracy** | **Sensitivity** | **specificity** | **PPV** | **NPV** | **F1 score** | **Kappa** |
| --- | --- | --- | --- | --- | --- | --- | --- | --- | --- |
| Original | 0.932(0.889-0.975) | 0.739(0.671-0.806) | 0.805(0.737-0.873) | 0.823(0.767-0.879) | 0.775(0.615-0.935) | 0.908(0.842-0.974) | 0.613(0.523-0.704) | 0.860(0.810-0.911) | 0.538(0.381-0.695) |
| PCA | 0.950(0.916-0.985) | 0.730(0.692-0.768) | 0.863(0.801-0.925) | 0.847(0.765-0.929) | 0.920(0.856-0.984) | 0.964(0.934-0.993) | 0.725(0.607-0.843) | 0.896(0.845-0.947) | 0.701(0.580-0.822) |

**Supplementary Figure S1. Confusion matrix heatmap.(** Suffix “1” indicates the original-variable group, whereas suffix “2” indicates the PCA group. **(a)** Train set. **(b)** Test set.)

**Supplementary Table S3. Comparative diagnostic performance of seven machine learning classifiers using internal nested cross-validation on the primary cohort.**

| **Classifications** | **AUC** | **cutoff** | **Accuracy** | **Sensitivity** | **specificity** | **PPV** | **NPV** | **F1 score** | **Kappa** |
| --- | --- | --- | --- | --- | --- | --- | --- | --- | --- |
| **LASSO + PCA (6 PCs)** | | | | | | | | | |
| Logistic | 0.940 (0.837-1.000) | 0.717(0.684-0.751) | 0.815(0.749-0.881) | 0.79(0.710-0.869) | 0.89(0.817-0.963) | 0.95(0.917-0.983) | 0.627(0.521-0.732) | 0.858(0.803-0.914) | 0.598(0.468-0.727) |
| XGBoost | 0.921 (0.825-0.989) | 0.818(0.798-0.837) | 0.893(0.823-0.964) | 0.903(0.844-0.961) | 0.875(0.753-0.997) | 0.949(0.895-1.003) | 0.778(0.662-0.894) | 0.924(0.872-0.976) | 0.743(0.584-0.902) |
| RandomForest | 0.933 (0.832-1.000) | 0.69(0.670-0.710) | 0.905(0.845-0.965) | 0.912(0.863-0.961) | 0.885(0.764-1.006) | 0.96(0.917-1.002) | 0.787(0.662-0.911) | 0.934(0.893-0.975) | 0.763(0.607-0.919) |
| SVM | 0.969 (0.910-1.000) | 0.701(0.612-0.789) | 0.893(0.839-0.947) | 0.887(0.844-0.929) | 0.91(0.817-1.003) | 0.965(0.927-1.004) | 0.747(0.654-0.840) | 0.924(0.885-0.963) | 0.744(0.616-0.871) |
| MLP | 0.641 (0.350-0.903) | 0.491(0.478-0.504) | 0.494(0.444-0.544) | 0.361(0.299-0.423) | 0.86(0.713-1.007) | 0.905(0.825-0.985) | 0.319(0.268-0.371) | 0.506(0.443-0.570) | 0.14(0.041-0.239) |
| DecisionTree | 0.809 (0.687-0.903) | 1.0(1.000-1.000) | 0.853(0.758-0.947) | 0.904(0.848-0.960) | 0.715(0.466-0.964) | 0.907(0.828-0.986) | 0.675(0.440-0.911) | 0.902(0.842-0.963) | 0.592(0.309-0.875) |
| GNB | 0.901 (0.751-1.000) | 0.631(0.567-0.695) | 0.75(0.690-0.810) | 0.775(0.683-0.867) | 0.69(0.562-0.818) | 0.88(0.836-0.924) | 0.564(0.448-0.681) | 0.815(0.762-0.868) | 0.425(0.316-0.533) |
| **Original LASSO (22 vars)** | | | | | | | | | |
| Logistic | 0.993 (0.974-1.000) | 0.624(0.578-0.670) | 0.947(0.920-0.974) | 0.96(0.926-0.995) | 0.91(0.837-0.983) | 0.969(0.945-0.994) | 0.908(0.830-0.987) | 0.963(0.945-0.982) | 0.864(0.794-0.934) |
| XGBoost | 0.980 (0.940-1.000) | 0.889(0.876-0.903) | 0.918(0.872-0.964) | 0.912(0.852-0.971) | 0.935(0.870-1.000) | 0.976(0.951-1.000) | 0.825(0.720-0.930) | 0.94(0.904-0.975) | 0.809(0.706-0.911) |
| RandomForest | 0.985 (0.953-1.000) | 0.725(0.703-0.747) | 0.917(0.868-0.966) | 0.904(0.835-0.973) | 0.955(0.896-1.014) | 0.985(0.965-1.005) | 0.819(0.708-0.930) | 0.938(0.900-0.977) | 0.812(0.708-0.916) |
| SVM | 0.996 (0.985-1.000) | 0.566(0.505-0.627) | 0.959(0.929-0.989) | 0.969(0.943-0.994) | 0.935(0.847-1.023) | 0.978(0.948-1.008) | 0.918(0.852-0.985) | 0.972(0.952-0.992) | 0.892(0.810-0.973) |
| MLP | 0.659 (0.361-0.944) | 0.826(0.803-0.848) | 0.523(0.441-0.605) | 0.41(0.309-0.512) | 0.84(0.715-0.965) | 0.872(0.777-0.967) | 0.342(0.286-0.399) | 0.544(0.443-0.646) | 0.173(0.056-0.290) |
| DecisionTree | 0.859 (0.681-0.988) | 1.0(1.000-1.000) | 0.881(0.824-0.939) | 0.912(0.858-0.966) | 0.805(0.694-0.916) | 0.928(0.886-0.970) | 0.782(0.655-0.910) | 0.918(0.878-0.958) | 0.703(0.559-0.848) |
| GNB | 0.939 (0.835-1.000) | 0.354(0.284-0.424) | 0.875(0.834-0.916) | 0.912(0.863-0.961) | 0.775(0.623-0.927) | 0.926(0.881-0.971) | 0.777(0.661-0.893) | 0.915(0.888-0.942) | 0.67(0.545-0.794) |

**Supplementary Figure S2.** Spearman correlation heatmap of the initial 38 candidate multimodal variables. Darker colors (red for positive, blue for negative) represent high correlation coefficients, indicating dense clusters of severe multicollinearity (e.g., among basic gait metrics and gait cycle phases).

**Supplementary Figure S3.** Spearman correlation heatmap of the 22 features retained after LASSO selection. The matrix illustrates a substantial reduction in dense collinear clusters, demonstrating LASSO's efficacy in pruning redundant variables while preserving clinically intuitive markers for subsequent PCA.

**Supplementary Table S4.** Diagnostic performance of the finalized PCA-Logistic framework in early (Hoehn and Yahr stages 1–2) and moderate (Hoehn and Yahr stages 3) Parkinson's disease subgroups.

|  | **AUC** | **cutoff** | **Accuracy** | **Sensitivity** | **specificity** | **PPV** | **NPV** | **F1 score** | **Kappa** |
| --- | --- | --- | --- | --- | --- | --- | --- | --- | --- |
| PD(H&Y 1-2)vs. HC | 0.928(0.872-0.985) | 0.683(0.634-0.732) | 0.819(0.748-0.889) | 0.817(0.731-0.904) | 0.830(0.650-1.010) | 0.931(0.861-1.002) | 0.708(0.575-0.840) | 0.860(0.806-0.914) | 0.595(0.427-0.763) |
| PD(H&Y 3)vs. HC | 0.950(0.875-1.025) | 0.336(0.162-0.509) | 0.907(0.842-0.972) | 0.850(0.700-1.000) | 0.940(0.856-1.024) | 0.917(0.805-1.028) | 0.940(0.880-1.000) | 0.847(0.744-0.950) | 0.787(0.644-0.930) |
